# Current Applications for the Use of Extracorporeal Carbon Dioxide Removal in Critically Ill Patients

**DOI:** 10.1155/2016/9781695

**Published:** 2016-02-04

**Authors:** Luigi Camporota, Nicholas Barrett

**Affiliations:** Department of Adult Critical Care, Guy's and St Thomas' NHS Foundation Trust, King's Health Partners, St Thomas' Hospital, 1st Floor East Wing, Westminster Bridge Road, London SE1 7EH, UK

## Abstract

Mechanical ventilation in patients with respiratory failure has been associated with secondary lung injury, termed ventilator-induced lung injury. Extracorporeal venovenous carbon dioxide removal (ECCO_2_R) appears to be a feasible means to facilitate more protective mechanical ventilation or potentially avoid mechanical ventilation in select patient groups. With this expanding role of ECCO_2_R, we aim to describe the technology and the main indications of ECCO_2_R.

## 1. Introduction

Respiratory failure—a condition in which the respiratory system is unable to maintain adequate gas exchange to satisfy metabolic demands—is the most common cause of admission to critical care, and because of the increase of life expectancy in industrialized countries, respiratory diseases will represent the third most common cause of death by 2025. An important syndrome leading to respiratory failure in critically ill patients is the acute respiratory distress syndrome (ARDS), which leads to poor lung function with hypoxaemia, hypercapnia, and low respiratory system compliance.

In these conditions, mechanical ventilation is often able to provide adequate oxygenation and CO_2_ removal. However, the improvement of gas exchange commonly occurs at the expense of a secondary injury to the lung (ventilator-induced lung injury or VILI) due to inhomogeneous lung overdistension. VILI can lead to the release of inflammatory mediators that reach other organs causing multiple organ failure [1]. This has led to the concept of protective mechanical ventilation with limited tidal volume and plateau pressure. Application of protective ventilator strategies is associated with decreased serum cytokine levels [2], decreased extra-pulmonary organ dysfunction [3], and decreased mortality [4].

In the last 15 years it has become evident that targets of mechanical ventilation traditionally considered “protective” are often unable to offer lung protection particularly in the more severe cases of lung injury. This has led to the concept of an “ultraprotective” ventilation strategy which employs even lower tidal volumes, a lower respiratory rate, lower driving pressures, and lower plateau pressures while maintaining an adequate mean airway pressure to avoid a reduction in functional residual capacity.

Ultraprotective ventilation strategies are likely to lead to hypercapnia and its deleterious consequences including systemic and cerebral vasodilatation, cardiovascular depression, arrhythmia, and pulmonary vasoconstriction with an increase in pulmonary arterial pressure. Acute pulmonary hypertension increases RV afterload and causes acute cor pulmonale which is associated with high mortality rates [5]. The need to correct hypercapnia without exposing the lung to mechanical trauma has resulted in a renewed interest in extracorporeal technologies that facilitate extracorporeal CO_2_ removal (ECCO_2_R).

## 2. Definition

ECCO_2_R is a technique of partial respiratory support that achieves removal of CO_2_ from the blood through a low blood flow (0.4–1 L/min) extracorporeal circuit, without significant effect on blood oxygenation. This is in comparison to extracorporeal membrane oxygenation which uses blood flows of 3–7 L/min to provide total respiratory support with significant oxygenation and CO_2_ removal. In many expert centres the devices used to provide ECMO and ECCO_2_R support are the same and given that the key difference is blood flow rates, there is considerable overlap between the two techniques. Indeed, some authors refer to low-flow ECMO rather than ECCO_2_R in order to acknowledge this continuum. Through ECCO_2_R a proportion of the total CO_2_ production is cleared to allow reduction of mechanical ventilation and allow “lung rest” [6]. Although the original purpose for ECCO_2_R was to provide additional CO_2_ clearance in patients with severe ARDS to allow reduction in tidal volumes and inspiratory pressures, its applications are extending to include patients with Chronic Obstructive Pulmonary Disease (COPD) and as a bridge to transplant or in order to facilitate thoracic surgery.

## 3. ECCO_**2**_R Gas Exchange Physiology

The oxygenation of blood and the removal of carbon dioxide (CO_2_) are physiologically different. Oxygen is mainly transported bound to haemoglobin, rather than being dissolved, and the mixed venous blood generally has high saturation (65–70%), which limits the amount of oxygen per litre that can be added to the blood perfusing the natural or the extracorporeal lung. This makes blood oxygenation dependent on blood flow (on average 4–7 L/min). In contrast, CO_2_ exchange depends on ventilation or, in the case of a membrane, gas flow (termed sweep gas flow). The sweep gas contains little or no CO_2_ and is passed through the membrane on the other side of a semipermeable membrane to the blood, thereby creating a diffusion gradient, which allows CO_2_ removal. By using a sweep gas of up to 100% oxygen at high flows, the gradient in partial pressure of oxygen and CO_2_ across the membranes separating the blood from gas can be significantly higher than the gradient across the capillary and alveolar wall in the native lungs.

Considering that 1 L of blood is transported around 500 mL of CO_2_ (double the CO_2_ production per minute, about 200–250 mL/min), in a perfectly efficient system a flow of 0.5 L/min would be sufficient to remove all of the CO_2_ produced [3, 7–9]. However, in practice CO_2_ removal at any given blood flow depends upon the gas flow and blood CO_2_ content and haemoglobin [10] as well as the efficiency of the gas exchange membrane. These limit the amount of CO_2_ removed from the blood and ECCO_2_R is usually able to remove up to 25% of carbon dioxide production [6]. Blood flow is of course also important and CO_2_ removal also varies with changes in blood flow through the device [4]. Apart from the fact that CO_2_ removal is proportional to the PaCO_2_, sweep gas flow, and blood flow, it also exhibits biphasic removal kinetics, exemplified by the initial rapid decline in PaCO_2_ secondary to removal of the dissolved CO_2_ and then followed by a more steady removal of CO_2_, liberated from bicarbonate [11].

## 4. Description of ECCO_**2**_R Systems

ECCO_2_R systems vary in characteristics, technology, and ability of gas exchange. They range from renal dialysis systems (low blood flow and low priming volume) to partial extracorporeal support (ECCO_2_R) up to systems capable of full ECMO support with the capability of removing all CO_2_ production and providing flows high enough to increase oxygen delivery and therefore provide blood oxygenation. An ECCO_2_R circuit consists of a percutaneously placed drainage cannula placed in a large central vein (or artery), a membrane lung, and a return cannula into the venous system. In the case of arteriovenous (AV) systems, the patient's blood pressure provides the driving pressure across the membrane. Venovenous systems require a pump to be placed within the circuit.

## 5. Access Cannulae

Access to the circulation is gained either through separate arterial and venous cannulae (AV systems) or by using double-lumen cannulae (VV systems).

The AV system requires two single-lumen wire-reinforced cannulae, one in the femoral artery to access blood and one in the femoral vein to return the blood to the venous side. The venous cannula is usually larger than the arterial cannula in order to reduce resistance to blood flow. The key disadvantage of the AV approach is the need for arterial cannulation with the potential side effects of arterial injury and limb ischaemia. In reported series distal ischaemia occurs in 11–24% of cases [12, 13]. The risk of ischaemia relates directly to the diameter of the arterial cannula. It is recommended that ultrasound of the artery takes place prior to cannulation to ensure that the arterial lumen is at least 1.5 times the size of the arterial cannula. Arterial injury is reported to occur in 7.5%–10% of cannulation cases [12, 14] for ECCO_2_R and is thought to relate to operator experience, patient factors including peripheral vascular disease, and size of arterial cannula. In the AV approach, flow is of course directly related to arterial cannula diameter and the risk of inadequate blood flow has to be balanced against the risk of arterial injury and limb ischaemia.

Cannulae for VV ECCO_2_R are conceptually identical to dialysis catheters. They are wire-reinforced, double-lumen coaxial cannulae between 13 and 19 Fr. Some cannulae have heparin coatings to reduce the risk of thrombosis. They can be placed in any large central vein although the jugular and femoral approaches are most commonly used [15]. The cannulae used for ECCO_2_R have the same complications as any central venous cannula, including risk of vessel perforation leading to bleeding or damage to surrounding structures, for example, pneumothorax or arterial injury [16].

Regardless of the approach chosen, cannulae are inserted using a percutaneous Seldinger technique. Ultrasound has been shown to reduce the risk of complications relating to central venous and ECMO cannula insertion and it would seem prudent to use ultrasound guidance for ECCO_2_R. Similarly it would seem prudent to observe strict aseptic technique during cannula insertion as this has been demonstrated to reduce complications relating to central venous cannula insertion. Not all cannulae are heparin bonded and either flushing with a heparinized saline solution or systemic anticoagulation with heparin will be required at the time of cannulation to prevent thrombosis of the cannula.

## 6. The Membrane Lung

The concept of placing a barrier between blood and air began with the observation that gas exchange occurred across cellophane tubing in haemodialysis machines [7, 17]. Indeed the membrane lung is conceptually very similar to modern haemodialysis filters using capillary tubules to carry blood through the oxygenator and separate tubules to carry the sweep gas. Recently, nonmicroporous poly-4-methyl-1-pentene (PMP) has been used; it provides superior gas exchange, better biocompatibility, and lower resistance and is less susceptible to plasma leak [7]. Modern membrane lungs achieve adequate gas exchange with surface areas of 1 to 3 m^2^. The gas exchange membrane is connected to air or oxygen which acts as a “sweep gas” to remove CO_2_ that has diffused out of the patient's blood. The flow rate of oxygen is increased in a stepwise fashion up to a maximum of 12 L/min.

## 7. The Pump

Blood flows through the ECCO_2_R circuits can occur in two ways.

1. The first one is using the patient's own arterial blood pressure (*pumpless systems*), where arterial blood is accessed through an arterial cannula and returned to the venous system via a venous cannula. These systems (arteriovenous, or AV ECCO_2_R) have the advantage of not requiring a mechanical pump. They therefore tend to be simpler and cheaper but have the disadvantage of requiring arterial cannulation with the potential side effects of arterial injury and limb ischaemia. The AV ECCO_2_R circuit introduces a low resistance iatrogenic shunt between the arterial and venous systems thereby reducing systemic vascular resistance and a compensatory increase in cardiac output to maintain systemic arterial perfusion pressure. In addition, the proportion of the cardiac output that flows through the ECCO_2_R system is not involved in peripheral perfusion; hence the patient's effective cardiac output is reduced. Furthermore, for optimal functioning, a mean arterial pressure of 70 mmHg and a cardiac index >3 L/min/m^2^ are required [18]. For that reason cardiac failure, severe haemodynamic instability, and severe peripheral vascular disease are a contraindication to AV ECCO_2_R.
2. Alternatively, blood flow can be achieved through a* mechanical pump*. The pump and the membrane can be separate components or can form a single console. Pumps can be* roller or peristaltic* (older systems) or rotary pumps, which can be “diagonal” or “centrifugal” where a rotating impeller creates a suction vortex that draws blood into the centre of the pump and propels it outwards from the outlet.

## 8. Experimental Evidence for Efficacy of CO_**2**_ Removal

Regardless of whether the AV or VV approach is used, ECCO_2_R devices can remove enough CO_2_ to allow a 50% reduction in minute alveolar ventilation [19], with significant reduction in PaCO_2_ and consequent reduction in pulmonary artery pressure and improvement in RV-arterial coupling even with flows as low as 0.6–0.7 L/min and average sweep gas flow of 8 L/min [20]. Livigni et al., in an adult sheep model, were able to obtain a constant removal of arterial CO_2_, with an average 20% reduction in CO_2_, with extracorporeal blood flow of 300 mL/min (around 5% of cardiac output) [21]. Other animal studies have demonstrated that the devices can maintain consistent CO_2_ removal for over 7 days using a low-flow VV approach [22]. Novel methods to maximize CO_2_ removal, such as regional blood acidification which increases the bioavailability of CO_2_ by unbinding it from the bicarbonate ion in circulating blood, are also under investigation [23, 24].

## 9. Clinical Evidence for Efficacy of CO_**2**_ Removal

In the mid-late 1970s Kolobow, Gattinoni, and Pesenti pioneered the use of venovenous ECCO_2_R for partial-to-total CO_2_ removal to allow low-frequency ventilation and lung rest [25–27]. In addition, these authors showed that the removal of one-third of the basal CO_2_ production through the extracorporeal circuit at flows of 400–600 mL/min allowed reduction of tidal volumes and the switch to noninvasive ventilation [28, 29]. Early clinical trials, however, did not show positive outcomes and the complication rate—particularly bleeding—was elevated [30–33]. In 1980, Gattinoni et al. showed that ECMO with a blood flow as low as 1.3 L/min drastically reduced ventilation needs (respiratory frequency of 2-3 breaths/min) thus limiting ventilator-induced lung injury [27]. A subsequent observational study of 23 patients with severe ARDS [34] demonstrated the ability of ECCO_2_R to allow reduction in respiratory rate and inflation pressures while maintaining CO_2_ clearance and supporting oxygenation. Bleeding was significant in this series and was the reported cause of death in 4/23 patients.

More recent series in humans have demonstrated consistent evidence that PaCO_2_ can be reduced and arterial pH due to respiratory acidosis improved using ECCO_2_R [35]. AV ECCO_2_R has also been shown to reduce minute ventilation in an uncontrolled cohort of 159 patients over 10 years showed [18]. Similarly VV ECCO_2_R can effectively reduce PaCO_2_ in patients with ARDS and can facilitate a reduction in both tidal volume and airway pressures [7, 9].

## 10. Current Evidence

The current evidence supporting applications for ECCO_2_R is extremely limited. Most published data is at the level of observational, retrospective case series. Consequently there is inherent bias including selection bias and complications and outcomes need to be interpreted with an understanding that the case series mainly originate from expert centres. At present ECCO_2_R should be considered a research tool, rather than an accepted clinical procedure. There is a clear need for further robust research, particularly prospective, randomized, controlled studies.

## 11. ARDS

Besides the general improvement in the standard of care provided in the intensive care units (ICUs), the only specific management that has consistently been shown to reduce mortality in ARDS is the provision of mechanical ventilation with static inspiratory pressures (plateau pressure) of less than 30 cm H_2_O and low tidal volumes normalised to predicted body weight (PBW) according to a concept known as “lung-protective ventilation” (LPV). This strategy has been shown to reduce ventilator-induced lung injury (VILI) and is linked to improvements in short and long term outcomes for the majority of patients [36].

However, despite this global improvement in survival, there is a cohort of patients with severe hypoxaemia (PaO_2_/FiO_2_ < 13.3 kPa) and hypercapnia (leading to a pH < 7.20) who offer a significant therapeutic challenge. This group of patients, even when managed with optimal recruitment and LPV, have a significantly higher mortality than patients with a higher PaO_2_/FiO_2_ ratio or lower PaCO_2_ for any given minute ventilation [37–39]. It is likely that the identification of this subgroup of patients with “severe” ARDS will be made more explicit in the new “Berlin definition of ARDS” [40], allowing for targeted therapeutic interventions and further clinical studies.

One of the potential rationales for the poorer outcomes in patients with more severe ARDS is that, due to the extent of lung consolidation and alveolar injury, the available lung volume is small. Hence the use of conventional low tidal volume ventilation (6 mL/Kg ideal body weight) when administered into a smaller available lung volume will yield excessive lung strain. Terragni et al. [41] demonstrated that up to one-third of patients receiving LPV had evidence of tidal hyperinflation and hence lung injury. One of the reasons for maintaining a conventional lung-protective ventilation approach is to limit the hypercapnia and consequent respiratory acidosis that will develop with very low minute ventilation. Although permissive hypercapnia has been advocated, hypercapnia may cause significant physiological instability including pulmonary hypertension and right ventricular failure leading to a global low cardiac output state. In these patients, the addition of ECCO_2_R may allow control of hypercapnic respiratory acidosis and facilitate ultraprotective ventilation thereby limiting end-inspiratory lung stretch. It is possible that this approach may improve patient outcome.

In 1994, Brunet et al. used extracorporeal CO_2_ removal combined with low-frequency positive pressure ventilation (ECCO_2_R-LFPPV) in severe ARDS. They showed that ECCO_2_R improved gas exchange and prevented lung overinflation [42]. However, the same year Morris et al., in a randomized trial, showed that in patients with severe ARDS full ECCO_2_R (meaning 100% CO_2_ removal) compared to conventional mechanical ventilation had a lower mortality of 33% versus 42% for the control group which failed to achieve statistical significance. The authors did not recommend extracorporeal support as a therapy for ARDS outside controlled clinical trials [43]. Subsequent studies have confirmed efficacy of ECCO_2_R in removing CO_2_ allowing reduction in tidal volumes to and below 4 mL/kg [44] but with high mortality and complications [12, 45]. Similarly, Zimmermann et al. showed that when ECCO_2_R was used as rescue in more severe ARDS (PaO_2_/FiO_2_ ratio <100 mmHg and/or pH < 7.25 after a 24-hour period of optimized ventilation) it achieved marked removal in arterial carbon dioxide allowing a rapid reduction in tidal volume (< or = 6 mL/kg) and inspiratory plateau pressure. Adverse events occurred in six patients (11.9%) [13]. The high mortality and disappointing effect of ECCO_2_R in the early use of ECCO_2_R were likely to be due to the complex extracorporeal systems with high flow resistances and large surface areas (3.5 m^2^), the use of occlusive roller pumps (with high haemolysis rate), and a less biocompatible membrane requiring high anticoagulation levels. In addition mechanical ventilation was in the pre-ARDSNet era and employed high tidal volumes and peak pressures.

Terragni et al. used VV ECCO_2_R in 32 ARDS patients to facilitate “ultraprotective” ventilation of 4 mL/kg [46]. VV ECCO_2_R treated the hypercapnic acidosis and allowed the plateau pressure to be reduced to 25 cm H_2_O to deliver 4 mL/kg ideal body weight tidal volume. Importantly the reduction in tidal volume did lead to tidal derecruitment and higher oxygen fraction; consequently there was a need for higher levels of positive end-expiratory pressure [PEEP] to maintain lung recruitment and functional residual capacity. The reduction in airway pressures seems to have reduced pulmonary inflammation as demonstrated by a reduction in bronchoalveolar inflammatory cytokines. In the prospective randomized “Xtravent-study,” Bein et al. [47] demonstrated that use of very low tidal volumes (3 mL/kg PBW) combined with ECCO_2_R with an arteriovenous configuration was safe and beneficial in patients with severe ARDS in terms of 28 and 60 ventilator-free days, but not mortality. A* post hoc* analysis showed increase in ventilation-free days at 60 days in patients with severe hypoxaemia (PaO_2_/FiO_2_ < 20 kPa) [47]. A recent systematic review of 14 studies (495 patients, two RCTs and 12 observational studies) with equal split between AV and VV ECCO_2_R showed that ECCO_2_R was feasible, facilitating the use of lower tidal volume ventilation [16], and* post hoc* analysis of data shows an increase in ventilator-free days in more severe ARDS although there has been no demonstrable mortality benefit to date.

Therefore, although the addition of ECCO_2_R to ultraprotective ventilation is appealing for patients with moderate-to-severe ARDS, at this time the effect of this approach on survival remains inconclusive [15, 42, 45]. Clearly the potential risk-benefit relationships still need to be defined, with the advantages of tidal volume/airway plateau pressure minimization being balanced against these risks associated with ECCO_2_R and derecruitment [16]. Finally we do not currently know which patient cohort is the key population to target. Patients with more severe respiratory failure (PaO_2_/FiO_2_ < 20 kPa) may be an appropriate population of patients, as, according to UK Intensive Care National Audit and Research Centre (ICNARC), this cohort has a 40% ICU mortality and around 50% hospital mortality. Clearly further research is required before this approach can be advised outside of clinical trials.

## 12. COPD

Patients who present with an acute hypercapnic respiratory failure due to a severe exacerbation of COPD often require hospitalization and noninvasive respiratory support (NIV) [5]. However, NIV fails to improve 25–50% of COPD patients who then require invasive positive pressure ventilation [48, 49]. These patients have prolonged weaning, and in-hospital mortality is as high as 25–39% [3, 6–9, 52]. Of concern, the mortality for patients who require invasive MV after failing NIV has been shown to be higher than those who are treated at the outset with invasive MV [6]. It is not clear why this is the case; however ventilator associated pneumonia is common in COPD patients requiring invasive mechanical ventilation and this has been shown to be an independent predictor of increased ICU mortality with mortality as high as 60–64% and VAP [50, 51].

In severe COPD exacerbations, high airway resistance, ventilation/perfusion mismatch, dynamic hyperinflation, and increased work of breathing with increased CO_2_ production lead to hypercapnia. Given the outcomes of patients requiring mechanical ventilation its avoidance in this population is potentially of clinical benefit. For this reason, ECCO_2_R is being considered as an adjunctive therapy to NIV to facilitate the withdrawal of NIV, avoid intubation, or facilitate early extubation. The feasibility of using venovenous ECCO_2_R for acute hypercapnic respiratory failure due to COPD exacerbations has been demonstrated in several recent cases and cohort studies [52–55].

A recent retrospectively propensity matched cohort study found that AV ECCO_2_R was able to consistently reduce PaCO_2_, improve respiratory acidosis, and reduce respiratory rate in 21 patients suffering from acute hypercapnic respiratory failure (mainly COPD) who were failing NIV [56]. In this retrospectively matched cohort study 90% of the patients treated with AV ECCO_2_R did not require intubation and invasive mechanical ventilatory support, and there was a trend in this group towards a reduced length of hospital stay, but not mortality.

Another recent study by Burki and colleagues reported on VV ECCO_2_R using a dual-lumen venous catheter in COPD patients with hypercapnic respiratory failure. Three groups of patients were studied: patients with a high likelihood of requiring intubation; patients who had failed two weaning attempts from continuous NIV support and did not wish to be intubated; or patients already on invasive ventilation to assist weaning in them [54]. PaCO_2_ and pH improved within 6 hours and there were minimal major complications of the technique. All patients in whom the goal was intubation avoidance or weaning from NIV achieved remained ventilator-free and separated from NIV. The approach was less successful in patients where early extubation was the goal in this study. However a feasibility study by Abrams et al. [52] enrolled five patients with acute respiratory acidosis in the setting of COPD exacerbations who had failed NIV and required IMV and were initiated on ECCO_2_R to facilitate endotracheal extubation and mobilization. All five patients were successfully extubated within 24 h (median duration, 4 h) and ambulating within 48 h of ECCO_2_R support. Furthermore dyspnea improved as the respiratory acidosis resolved. This approach of using ECCO_2_R after NIV failure was also used successfully in a small study by Roncon-Albuquerque et al. [53]. Finally there has been a recent retrospective cohort study using historical controls reported from Italy where 25 patients at high risk of NIV failure who received ECCO_2_R via a 14 Fr dual-lumen cannula in the femoral vein had lower intubation rates (HR 0.27) and a lower mortality [57]. Thirty-six percent of patients experienced device malfunctions and 12% of patients had bleeding complications, including one vessel perforation.

In summary the arguments for ECCO_2_R in exacerbations of COPD are compelling, although the evidence remains relatively weak. In patients who are failing NIV and who do not want intubation but also do not want palliative care ECCO_2_R seems a reasonable approach [58].

## 13. Thoracic Surgery

A few case reports show that ECCO_2_R has been successfully applied to patients who underwent elective or emergency thoracic surgery, to allow surgery in patients who would not have tolerated one-lung ventilation [59].

## 14. Bridge to Transplant

A further group of patients—those awaiting lung transplantation who develop life-threatening hypercapnia—may benefit from ECCO_2_R as bridge to transplantation (LTX). The concept behind using ECCO_2_R as a bridge to transplantation is that it is known that when patients waiting for lung transplantation have an acute deterioration requiring mechanical ventilation, their mortality is substantially increased when compared with those patients who do not require mechanical ventilation. Consequently, clinicians have used ECCO_2_R in selected hypercapnic patients in an attempt to avoid mechanical ventilation, in the hope that this will improve the likelihood of survival. The possible advantages of ECCO_2_R are the avoidance of intubation and mechanical ventilation and their potential adverse sequelae, continuation of physiotherapy and allowing the patient to remain autonomous. ECCO_2_R does have significant potential problems, including the need for anticoagulation and significant uncertainty as to the outcomes of patients awaiting transplantation supported with ECCO_2_R. To date there have been no randomized controlled trials performed utilizing this approach.

In a study of 20 patients, the most common underlying diagnoses were bronchiolitis obliterans syndrome, cystic fibrosis, and idiopathic pulmonary fibrosis. Hypercapnia and acidosis were effectively corrected in all patients within the first 12 h of ECCO_2_R therapy: nineteen patients (95%) were successfully transplanted. Hospital and 1-year survival were 75 and 72%, respectively [60]. AV ECCO_2_R devices have also been surgically implanted from the pulmonary artery to left atrium as a bridge to lung transplantation in patients with significant pulmonary hypertension [31]. Patients have similarly been successfully bridged to lung transplant using VV ECCO_2_R [44, 59–61]. The use of ECCO_2_R as a bridge to transplantation does appear promising in the case series reported to date; however significant further studies are required in order to confirm that this approach is appropriate and improves outcomes. Use of ECCO_2_R in patients awaiting transplantation should only be considered by expert centres with a balance of risk and benefit to the individual patient considered.

## 15. Conclusions

Technological advances in ECCO_2_R, together with the recognition that ventilation-induced lung injury can occur despite lung-protective ventilation, have created the opportunity for an extended role of partial extracorporeal CO_2_ removal. Future applications will involve smaller and more biocompatible ECCO_2_R systems, for patients with moderate-severe ARDS but also as a sole supportive modality in patients with hypercapnic respiratory failure. The potential complications of ECCO_2_R need to be evaluated when considering patients for extracorporeal support.
